# Putting the Screen in Screening: Technology-Based Alcohol Screening and Brief Interventions in Medical Settings

**DOI:** 10.35946/arcr.v36.1.07

**Published:** 2014

**Authors:** Sion Kim Harris, John R. Knight

**Affiliations:** Sion Kim Harris, Ph.D., is assistant professor in the Department of Pediatrics at Harvard Medical School and a research associate in the Department of Medicine, Boston Children’s Hospital, Boston, Massachusetts. John R. Knight, M.D., is associate professor in the Department of Pediatrics at Harvard Medical School; senior associate in the Department of Medicine; and associate in the Department of Psychiatry at Boston Children’s Hospital, Boston, Massachusetts.

**Keywords:** Alcohol use, abuse, and dependence, screening and brief intervention, medical setting, primary care, emergency room, adult, adolescent, pregnant women, technology, computer-based screening and brief intervention, literature review

## Abstract

Alcohol is strongly linked to the leading causes of adolescent and adult mortality and health problems, making medical settings such as primary care and emergency departments important venues for addressing alcohol use. Extensive research evidence supports the effectiveness of alcohol screening and brief interventions (SBIs) in medical settings, but this valuable strategy remains underused, with medical staff citing lack of time and training as major implementation barriers. Technology-based tools may offer a way to improve efficiency and quality of SBI delivery in such settings. This review describes the latest research examining the feasibility and efficacy of computer- or other technology-based alcohol SBI tools in medical settings, as they relate to the following three patient populations: adults (18 years or older); pregnant women; and adolescents (17 years or younger). The small but growing evidence base generally shows strong feasibility and acceptability of technology-based SBI in medical settings. However, evidence for effectiveness in changing alcohol use is limited in this young field.

Alcohol-related screening and brief interventions (SBIs) in medical settings have the potential to transform the treatment of alcohol misuse and prevent considerable alcohol-related harm (Babor and Higgins-Biddle 2001). Rapid screening and assessment tools allow health care providers to quickly assess the extent of patients’ alcohol use, identify those with problematic use, provide them with an immediate brief intervention, and refer patients with more severe alcohol use disorders to a substance abuse specialist when available. SBIs have proven effective for detecting potential alcohol problems and reducing the severity of problems in a wide range of populations and settings (Kaner et al. 2009; O’Donnell et al. 2014)—so much so that agencies focused on preventing and treating alcohol use, including the U.S. Preventive Services Task Force (USPSTF), the National Institute on Alcohol Abuse and Alcoholism (NIAAA), and the Substance Abuse and Mental Health Services Administration (SAMHSA), recommend that primary care and other medical settings expand their SBI use for patients ages 18 years and older (Moyer 2013; NIAAA 1995; SAMHSA 2011). Although the USPSTF cited insufficient evidence to recommend SBIs for adolescents (Moyer 2013), recognition of and evidence for the potential utility of SBIs for adolescents have been building in recent years (Harris et al. 2012; Mitchell and Gryczynski 2012; Pilowsky and Wu 2013), leading the American Academy of Pediatrics to recommend that all pediatricians use SBIs in their practices as part of routine care (American Academy of Pediatrics 2011).

Despite the push for using SBIs in medical settings, they remain underused. In a recent national survey of U.S. adults, only one in six (15.7 percent) respondents reported discussing alcohol use with a health professional in the past year, with State-specific estimates ranging from 8.7 percent to 25.5 percent (McKnight-Eily et al. 2014). The percentage was higher (34.9 percent), but still inadequate, among those with 10 or more binge-drinking episodes in the past month. An often-cited barrier to SBI implementation is lack of time (Van Hook et al. 2007; Wilson et al. 2011). Computer-facilitated SBI delivery may offer a solution for busy medical settings, allowing more widespread implementation. This article focuses on current-and emergingtechnology–facilitated SBI tools that have been evaluated in primary care, pediatric, and emergency department (ED) settings. We review studies of technology-based SBI as they relate to adults (18 years or older), pregnant women, and adolescents (17 years or younger), the primary patient populations in which alcohol SBIs have been implemented. The studies reviewed here come from a systematic electronic literature search conducted between February 2014 and December 2014 using PubMed and PsycINFO, as well as the reference lists of published studies and review articles. We summarize the characteristics of the studies, including population, design, and results, in the table.

## Value Added With Electronic SBIs

Technology-based SBIs could help increase the frequency and quality of SBI use in medical settings by enhancing efficiency and standardizing implementation. In terms of screening, touchscreen devices or standalone computers with Internet connections can allow patients to enter information in the waiting room prior to an appointment. Programs automatically score the screening results that staff can print or electronically transmit to practitioners. This reduces clinician time needed for administering and scoring a questionnaire during the visit. In addition, programs can be loaded with validated measures that improve the quality of screening and can automatically select appropriate questions according to the patient’s age and previous responses. Patients also may be more willing to disclose sensitive information to a computer than to a person (Butler et al. 2009; Turner et al. 1998), and integration of computerized screening results with electronic health records may boost screening and documentation rates (Anand et al. 2012).

Similarly, computer-facilitated brief intervention delivery has the potential advantages of greater standardization, lower cost, and greater ease of implementation compared with face-to-face delivery. As with screening, programs can automatically tailor intervention content to individual patients. Interventions vary based on the program, but, as with face-to-face SBIs, computer-based SBI tools often follow screening with personalized feedback that includes a summary of patients’ consumption patterns and risk status, a comparison of their consumption with recommended limits, estimated blood alcohol concentrations for their heaviest drinking occasion in the reported time frame, and a comparison between their consumption and consumption reported by others in their peer group. More extensive programs may incorporate intervention strategies based on principles of evidence-based face-to-face treatments, such as motivational interviewing (Miller and Rollnick 2012) and cognitive–behavioral therapy (Kadden et al. 1995).

Using technology for SBIs in medical settings may be especially valuable for reaching young people who are highly engaged with technology and nearly universal access to computers, cell phones, and the Internet (Madden et al. 2013; Marsch et al. 2007; Pew Research Center’s Internet and American Life Project 2014). Indeed, using technology-facilitated alcohol SBIs in medical settings to reach adolescents may be a powerful mechanism to reduce medical costs and gain productive years of life, since alcohol use disorders are strongly linked to the leading causes of adolescent and adult mortality, including motor-vehicle crashes and suicide.

This high level of online engagement has fueled a surge of interest in the potential of standalone Web-based SBI programs to address problematic alcohol use, particularly among college students. These programs provide a means to inexpensively reach people less likely to access traditional health services. Detailed reviews of research on these standalone online alcohol SBIs are provided in articles by Carroll and Cronce in this issue and suggest that, at least among college students and adults, these programs tend to yield small to moderate effects, which are greatest at followups less than 3 months, gradually declining to little or no effect by 12 months (Donoghue et al. 2014). The lack of interpersonal contact with these programs may contribute to lower participation rates and adherence over time (Murray et al. 2013; Naimi and Cole 2014; Postel et al. 2011). In addition, alcohol use is strongly linked to many physical and mental health problems, such as cancer, cirrhosis, and depression (National Center on Addiction and Substance Abuse 2011). Therefore, standalone programs are unlikely to obviate the need for SBIs in medical settings, which is the focus of this review.

## Medical Setting SBI for Adults

Twelve studies of varying design and stages of research (reported in 13 published papers) have examined computerized SBIs for adults in medical settings that include four studies in primary care (Bendtsen et al. 2011; Butler et al. 2003; Cucciare et al. 2013; Kypri et al. 2008), seven in EDs (Blow et al. 2006; Karlsson and Bendtsen 2005; Murphy et al. 2013; Neumann et al. 2006; Nilsen et al. 2009; Suffoletto et al. 2012; Trinks et al. 2010; Vaca et al. 2011), and one in a hospital outpatient department (Johnson et al. 2013) (see the table for study details). Half of the studies used a randomized design (Blow et al. 2006; Cucciare et al. 2013; Kypri et al. 2008; Neumann et al. 2006; Suffoletto et al. 2012; Trinks et al. 2010); one used a before-and-after design, with each clinic serving as its own control (Butler et al. 2003); and five are earlier-stage observational studies with small sample sizes (Bendtsen et al. 2011; Johnson et al. 2013; Karlsson and Bendtsen 2005; Murphy et al. 2013; Vaca et al. 2011). Generally, followup, where it existed, was short, with two studies following participants for 3 months, four for 6 months, and three for 12 months. The studies shared some common components.

### SBI Delivery Method

All but one study by Suffoletto and colleagues (2012), tested screening and/or brief intervention delivery on a tablet or desktop computer located in the medical setting. Suffoletto and colleagues (2012) delivered their intervention through weekly mobile text messages following patient discharge from the ED.

### Screening

All 12 studies used a self-administered computerized screener that assessed quantity and frequency of alcohol consumption and heavy episodic drinking (HED) episodes. Ten of the 12 studies (Butler et al. 2003; Cucciare et al. 2013; Johnson et al. 2013; Karlsson and Bendtsen 2005; Kypri et al. 2008; Murphy et al. 2013; Neumann et al. 2006; Suffoletto et al. 2012; Trinks et al. 2010; Vaca et al. 2011) used the Alcohol Use Disorders Identification Test (AUDIT) screening tool (Reinert and Allen 2002) or its shortened form, the AUDIT-C (Bush et al. 1998).

### Brief Intervention Delivery

Seven of the studies (Blow et al. 2006; Cucciare et al. 2013; Kypri et al. 2008; Neumann et al. 2006; Suffoletto et al. 2012; Trinks et al. 2010; Vaca et al. 2011) only provided the brief intervention portion of the SBI to patients who screened positive for risky drinking, typically defined as AUDIT-C scores of 4 or higher for men and 3 or higher for women, or AUDIT scores of 8 or higher. The other five studies (Bendtsen et al. 2011; Butler et al. 2003; Karlsson and Bendtsen 2005; Murphy et al. 2013; Johnson et al. 2013) provided a brief intervention regardless of alcohol use level.

### Brief Intervention Format

The brief interventions in 4 of the 12 studies (Bendtsen et al. 2011; Blow et al. 2006; Karlsson and Bendtsen 2005; Nilsen et al. 2009) were provided to patients using computer-generated printouts, whereas the rest were offline or Web-based computer programs. All but one computerized brief intervention consisted of a single session that lasted 10 to 20 minutes. The outlier examined both a single-dose Web-based brief intervention and a multi-dose version, where patients repeated the brief intervention at the 1and 6-month followups (Kypri et al. 2008).

### Brief Intervention Content

Nearly all of the brief interventions tested in these studies used at least some components of the FRAMES (Feedback, Responsibility, Advice, Menu of options, Empathy, Self-efficacy) model of brief intervention (Hester and Miller 1995). All the brief interventions in these studies provided feedback about the patient’s risk level, drinking pattern relative to recommended limits, advice and information to enhance motivation to avoid use, and suggestions for behavior change strategies, if applicable. Capitalizing on a key feature of computerization, most of the brief interventions automatically tailored feedback and information to patients’ screening results and other characteristics. That said, one of the randomized studies specifically examined the effect of tailored messages, compared with generic messages, either with or without clinician brief advice and found no significant effect of tailoring on alcohol consumption or related consequences after 12 months (Blow et al. 2006). Instead, patients who received brief advice from clinicians showed greater reductions in drinking than those who only received feedback from the computer SBI. Only one other study (Butler et al. 2003) included a printed report for the clinician with screening results and suggested brief intervention options. All other studies used technology-based self-guided brief intervention, with no explicit clinician involvement.

### Findings

Among the seven experimental or quasi-experimental trials (Blow et al. 2006; Butler et al. 2003; Cucciare et al. 2013; Kypri et al. 2008; Neumann et al. 2006; Suffoletto et al. 2012; Trinks et al. 2010), findings were mixed, with several reporting differences between the intervention and comparison conditions in follow-up outcomes and others not. Overall, the 12 studies suggested that using technology-based SBIs in medical settings is feasible and acceptable to patients but were not able to clarify whether they are effective.

### Primary Care

One controlled trial in a primary care setting (Kypri et al. 2008) found significant reductions in alcohol consumption scores and alcohol-related problems at both the 6- and 12-month followups among university health service patients in New Zealand who screened positive for alcohol problems and received a Web-based brief intervention, compared with patients who received a brochure. Two other trials (Butler et al. 2003; Cucciare et al. 2013) found reductions in alcohol consumption and related consequences out to 6 months, but the reductions were similar for both the standard care control and the computerized SBI groups. A fourth nonexperimental implementation study (Bendtsen et al. 2011) found that patients given access to a computerized SBI kiosk in a primary care clinic showed declines in heavy episodic drinking frequency at a 3-month followup. Patients referred to the SBI by a clinician, as opposed to those who self-initiated SBI use, showed a decline in weekly alcohol consumption. Without a control group, it is impossible to determine how much the decline is attributable to the SBI or some other confounder. That said, this study is unique in its examination of a computerized SBI system that routinely was offered at a primary care clinic, independent of a research study, showing that patients and clinicians are willing to use the system.

### EDs

Only two of the ED studies used a nonintervention control group. One study (Neumann et al. 2006), a large German trial of 1,139 sub-critically injured ED patients with at-risk drinking, found significantly reduced prevalence of at-risk drinking and alcohol consumption at both the 6-and 12-month followups for patients receiving computerized SBIs compared with those receiving standard care alone. Another, much smaller study (Suffoletto et al. 2012) conducted in three Pennsylvania EDs sent weekly text messages (TMs) to young-adult risky drinkers discharged from the EDs. The intervention group received TMs asking them to evaluate their drinking and providing them with information about setting alcohol consumption goals. Another group received TMs asking them to assess their drinking. A third group simply received TM notifications about the study’s 3-month followup. Participants in the goal-setting intervention significantly reduced hazardous drinking behavior, compared with participants in the control groups (Suffoletto et al. 2012). However, this study found the greatest change among those with the highest baseline drinking levels, suggesting potential regression to the mean, which is a statistical phenomenon where more extreme values in data tend to move spontaneously towards the mean over time as a result of a certain amount of natural variation (Barnett et al. 2005). The other two ED studies did not use nonintervention control groups. Instead, they compared different active interventions. Both found that all the interventions tested reduced weekly alcohol consumption and HED frequency (Blow et al. 2006; Trinks et al. 2010), as well as alcohol-related consequences (Blow et al. 2006). All ED studies excluded patients that were intoxicated, had a high blood alcohol concentration at time of recruitment, were suicidal, or were otherwise being referred to psychiatry, which may have excluded patients with the most severe alcohol problems.

## SBIs for Pregnant Women

Previous studies have shown the benefits of SBIs for addressing alcohol and drug use in pregnant women (Chang 2002; Ondersma et al. 2011). However, only one published randomized-controlled trial (Tzilos et al. 2011) has examined a computerized SBI for alcohol use during pregnancy. This early-stage randomized controlled trial in an urban prenatal care clinic included a convenience sample of 50 pregnant women that either screened positive on the T-ACE alcohol screening tool (Elliot and Hickam 1990; Sokol et al. 1989) or had drinking patterns before pregnancy that exceeded NIAAA drinking limits for women (NIAAA 2010). Participants randomly completed either the computerized SBI or an unrelated questionnaire. Those receiving the intervention gave it high marks for ease of use, likability, and respectfulness. Both intervention and control groups showed significant and equivalent reductions in drinking at the 1-month followup, although babies born to women in the intervention group had higher newborn birth weights.

More recently, Pollick and colleagues (2013) found high acceptability of, and user satisfaction with, a computerized brief intervention for alcohol use in pregnancy (C-BIAP) in a qualitative pilot study among 18 pregnant African-American women. Given the paucity of studies in this population, and that alcohol use in pregnant and parenting women additionally can cause secondary lifelong harm to the fetus or infant, more studies are critically needed to elucidate the utility of computerized strategies to enhance the efficient and effective implementation of alcohol SBIs in prenatal and antenatal clinics.

## Targeting Adolescents

Numerous studies suggest that computerized screening of adolescent patients for alcohol use problems is acceptable, feasible, and effective in medical settings (Chisolm et al. 2008; Harris et al. 2012; Olson et al. 2009; Ozer et al. 2005; Stevens et al. 2008). Using computerized alcohol screening can increase adolescent satisfaction with the medical encounter (Gadomski et al. 2014; Harris et al. 2012) and efficiently boost physician recognition of substance use issues and patient–physician dialogue around substance-use topics (Harris et al. 2012; Olson et al. 2009; Stevens et al. 2008). These findings may help to bolster the case for increased adolescent screening for alcohol in medical settings, where screening rates remain suboptimal (Hingson et al. 2013).

Few studies have tested integrated computerized alcohol SBIs in adolescents. In fact, only four trials, yielding eight published papers (Cunningham et al. 2009, 2012; Gregor et al. 2003; Harris et al. 2012; Louis-Jacques et al. 2014; Maio et al. 2005; Walton et al. 2010, 2014), support computerized alcohol SBIs as feasible, acceptable, and, in some cases, effective for reducing drinking or alcohol-related problems among adolescents seen in medical settings.

Three of the four studies (Cunningham et al. 2012; Maio et al. 2005; Walton et al. 2014) were randomized controlled trials conducted among adolescent ED patients in the United States. These studies compared adolescents receiving standard care with adolescents receiving an integrated computerized SBI that screened patients and then delivered an approximately 30-minute single-session, highly interactive, tailored brief intervention that reflected principles of motivational interviewing (MI) and the social cognitive theory of behavior change (Bandura 1977). One trial (Maio et al. 2005) implemented a universal brief intervention aimed at both preventing and reducing use in adolescents with minor injuries. The other two only provided the brief intervention for adolescents who reported drinking in the past 12 months (Cunningham et al. 2012) or that screened positive for risky drinking on the AUDIT-C (Walton et al. 2014). The latter two trials additionally compared a single-session, computer-delivered brief intervention with a therapist-delivered version that was similar in content (Cunningham et al. 2012; Walton et al. 2014).

Overall, these ED-based studies found no significant differences in alcohol consumption outcomes between the intervention and standard-care control groups during followup, but some did find that the computer-based SBIs influence other alcohol-related behaviors in certain populations:

- Maio and colleagues (2005) found in post hoc subgroup analysis a significant intervention effect on frequency of alcohol misuse and HED behaviors among adolescents admitting to having driven while impaired before entering the study. It may be that computerized brief interventions based on motivational enhancement approaches, like their face-to-face counterparts, tend to be more effective for individuals that have at least a certain level of substance use, or experience of negative consequences (Blow et al. 2009; Palfai et al. 2011; Spirito et al. 2004). Alternatively, those with greater use may be more subject to regression to the mean (Finney 2008).
- At a 6-month followup, Cunningham and colleagues (2009, 2012) found that their computerized and therapist-delivered brief interventions, which addressed peer violence and alcohol use (Walton et al. 2010) were associated with greater reductions in alcohol-related consequences, such as missing school because of alcohol use, compared with patients receiving the standard-care control. By the 12-month followup, patients receiving the therapist-delivered brief intervention maintained reductions in peer violence, but neither intervention continued to influence alcohol-related outcomes. The authors postulate that it may be difficult to address effectively more than one risk area with a brief intervention.
- Walton and colleagues (2014) examined the intermediate effects of a single-session, computerized or therapist-delivered brief intervention on psychological constructs hypothesized to be key moderators of behavior change. They were looking for the “active ingredients” that bring about change in adolescent risky drinkers. They found that, among 836 urban adolescent ED patients with risky drinking, those receiving either brief intervention significantly increased their perception that it was important to stop drinking, compared with adolescents receiving standard care. In addition, those receiving the therapist-delivered intervention increased their readiness to stop drinking. The analysis teased out two brief intervention components that had the strongest effect on these psychological outcomes, regardless of delivery mode: a review of personal strengths and suggested tools patients could use to reduce or stop drinking. Within the computer-delivered brief intervention, the components that most influenced outcomes were those that helped patients identify more benefits of behavior change, imagine sports activities that could be alternatives to alcohol use, and choose a goal to reduce or stop drinking. In contrast, the component of the therapist-delivered brief intervention that provided normative statistics/personalized feedback about current level of use was associated with negative effects on these cognitive outcomes. This study is ongoing and has yet to determine how these intermediate changes and brief intervention components connect to actual alcohol use and related consequences. However, it represents an important direction for future research into computerized SBI systems, such as the determination of the most effective ingredients, thus promoting the development of the most efficient and effective interventions possible.

The one adolescent trial of a computer-facilitated SBI conducted in a primary care setting involved several primary care clinics in the United States and the Czech Republic (Harris et al. 2012). The study utilized a before-and-after comparison design. Each clinic enrolled participants while providing standard care; then the clinic enrolled a comparison group of participants after implementing a computer-facilitated SBI system. The system consisted of three components:

- A pre-visit computerized screening using the CRAFFT behavioral health screening tool designed for children under age 21 (Knight et al. 2002);
- Immediate computer-delivered feedback to patients about their risk level, followed by several interactive pages of science-based and true-life information about substance-related health-risks and other harms; and
- Brief advice from a clinician during the primary care visit based on a printed provider report that suggested discussion points about substance use and related driving/riding risks tailored to each patient according to the screening results.

This multisite study found that U.S. adolescents, but not Czechs, had significantly reduced their alcohol use at the 3and 6-month followups, although reductions at 12 months were less robust. In addition, the computer-facilitated SBI reduced both drinking initiation and cessation in the U.S. sample (Harris et al. 2012), and the short-term cessation effect actually was largest among drinking youth with friends who drink or approve of drinking (Louis-Jacques et al. 2014). This study also found a significant intervention effect in both countries at the 3-month followup on prevalence of driving after drinking or riding with a driver who had been drinking (Harris et al. 2011).

Because the computer system used in this study was designed to be integrated into a face-to-face primary care visit, these findings cannot disentangle the relative effects of the computerized versus the face-to-face components of the brief intervention. To this end, studies in adolescents are needed that use a factorial design (such as the study by Blow et al. 2006) to test the relative efficacy of clinician advice versus the computerized component.

With only four trials (Cunningham et al. 2009, 2012; Gregor et al. 2003; Harris et al. 2012; Louis-Jacques et al. 2014; Maio et al. 2005; Walton et al. 2010, 2014), the evidence currently is insufficient to recommend computerized alcohol SBIs among adolescents in either EDs or primary care settings. More high-quality studies with randomized controlled designs and large sample sizes are needed, particularly in the primary care setting, which represents a key touch point with the health care system for adolescents where alcohol use can be detected early and where brief interventions are most likely to be effective. Alcohol and drug dependence are chronic, relapsing disorders with high treatment costs that most often begin during childhood. Given the relatively low risks and costs, and potential for benefit, of computerized prevention and early intervention, clinicians may wish to implement them as they become available.

## Discussion and Future Directions

Research on technology-facilitated SBIs in medical settings is in its infancy. As such, there remain many questions and methodological issues that researchers should address when evaluating these interventions.

### Special Populations

Although there is some evidence that the effectiveness of alcohol SBIs may be greater for people who have already experienced problems or negative consequences of drinking, it is unclear whether such programs are useful for patients with alcohol dependence (Saitz 2010). In addition, more studies should be conducted among pregnant women and adolescents, as well as in diverse racial and ethnic groups. Finally, studies should evaluate the effectiveness of Web-based alcohol SBI in high-risk, underserved, and remote populations, such as military personnel, American Indians, and Eskimo/Inuit, as such systems are particularly suited to access such hard-to-reach groups.

### Screening Validity

Evidence to date suggests that responses to computerized screening are reliable and comparable to other screening modes (McNeely et al. 2014; Thomas and McCambridge 2008; Williams et al. 2000). However, other studies suggest differences between the two modalities that researchers may want to consider as they design their programs. For example, some studies find that people are more likely to report more sensitive or stigmatized behaviors, such as illicit drug use or higher levels of alcohol consumption, on computer self-administered questionnaires compared with face-to-face interview (e.g., Beck et al. 2014; Butler et al. 2009; Perlis et al. 2004) or even self-administered paper-and-pencil questionnaires (Wright et al. 1998). Additionally, adolescents seem to be particularly sensitive to mode and context effects when reporting sensitive behaviors (Gfroerer et al. 1997; Turner et al. 1998; Wright et al. 1998). In fact, a study of adolescent primary care patients found that their reactions to computerized screening was highly associated with their level of trust in the data being kept secure and private and used only for health care (Chisolm et al. 2008). Other studies suggest that factors such as language (Butler et al. 2003) and gender (Neumann et al. 2004) also may affect computerized screening performance.

### Intervention Intensity

There is little evidence to date that the length of the intervention influences its effectiveness. No study in this review directly compared the effects of low-intensity to longer interventions, but there seemed to be no consistent pattern across trials indicating greater efficacy of longer interventions over shorter. A recent meta-analysis (Carey et al. 2012) of a computerized brief intervention targeting college students found that the effectiveness of the intervention was not affected by duration.

As for single-session versus multi-session interventions, the primary care study by Kypri and colleagues (2008) was the only trial reviewed here to compare the two directly. It found no increased benefit of additional brief intervention doses given at 1 and 6 months. This finding corroborates the conclusions of other reviews (Rooke et al. 2010; Donoghue et al. 2014; Kaner et al. 2007) that found no significant effect of the number of treatment sessions on the average effect size of computer-delivered and face-toface SBIs (Kaner et al. 2007). A more recent 2012 review of face-to-face SBI studies did find larger effect sizes for brief (less than 15 minutes each) multi-contact interventions, compared with very brief (up to 5 minutes) or brief (5 to 15 minutes) single-contact interventions (Jonas et al. 2012). Compared with face-to-face delivery, technology-based delivery modes, including via the Internet or cell phones, offer the advantage of relative ease and low cost of delivering multiple doses. Therefore, further exploration of the question of optimal number of doses is clearly warranted.

### Face-to-Face vs. Computerized Delivery

Another important question is whether self-guided computerized SBIs are as effective as face-to-face SBIs. Only four of the reviewed trials compared the two modalities. Two trials (Cunningham et al. 2012; Walton et al. 2014) directly compared a 35-minute therapist-delivered SBI and a self-guided computerized SBI provided to adolescent ED patients. Both modalities showed similar reductions in alcohol-related consequences and positive changes in psychological precursors to behavior change compared with a standard-care control (Cunningham et al. 2012; Walton et al. 2014). Other studies and reviews comparing face-to-face and technology-facilitated SBIs outside medical settings find an edge for face-to-face (Carey et al. 2012; Donoghue et al. 2014). It may be that combining face-to-face and technology-based SBI will be the most effective. Such a combination is easily accomplished in a medical setting where patients could complete a computerized portion of the alcohol SBI before a face-to-face encounter. This would screen and “prime” the patient to discuss the topic when meeting with the clinician and could increase clinician fidelity of brief intervention implementation by using “prompts” to guide the clinician. Although computers have certain logistical advantages, they cannot convey empathy, regard, and complex reflections, which represent some of the most important ingredients of brief motivational interventions (Miller and Rollnick 2012). Also, patients may put less attention, thought, and effort into completing a computerized brief intervention compared with a face-toface intervention (Walters and Neighbors 2011). Future research will benefit from examining a combination of face-to-face and computerized SBI delivery, as it may help to achieve larger and more enduring effects than self-guided computerized SBIs alone (White et al. 2010).

### Outcome Measures

In terms of what intervention studies measure, more need to consider alcohol-related outcomes other than consumption, including negative consequences and problems related to alcohol use such as school problems for adolescents, driving while impaired, traffic violations, and crashes and injuries. Among the studies reviewed here, not all examined these outcomes, yet, in the face-to-face alcohol SBI literature, intervention effects on alcohol-related consequences or risks often have been larger than on alcohol consumption (Newton et al. 2013; Wachtel and Staniford 2010; Yuma-Guerrero et al. 2012). Therefore, failure to measure such outcomes, which have great public health import, may be a missed opportunity to identify some key intervention benefits.

### Mediators and Moderators

There is a dearth of studies on mediators and moderators of the effects of computerized SBI in any setting and, in particular, within the small subset of studies examining these interventions within medical settings. Only one study (Walton et al. 2014) reviewed here attempted to elucidate the potential mechanisms and “active ingredients” underlying the effects of the computerized SBIs delivered to adolescents in an ED. Within the broader literature, the meta-analysis by Carey and colleagues (2012) found reduced computerized SBI effectiveness when the intervention included a decisional-balance or values-clarification exercise, identified high-risk situations, or included moderation strategies.

A few studies have found that certain patient characteristics, such as baseline stage-of-change or severity of alcohol involvement also may moderate the effectiveness of computerized SBIs. Among the studies reviewed here, Neumann and colleagues (2006) found greater intervention impact among patients who were contemplating changes in their drinking habits when they entered the study, and Vaca and colleagues (2011) found their SBIs to be more effective among patients reporting recent drinking and driving. The finding that an intervention may be more effective among individuals with more risky drinking behavior matches findings from a recent review of face-to-face alcohol/drug SBIs for adolescents seen in medical settings (Mitchell et al. 2013) and a study of a computerized SBI for college students (Carey et al. 2012).

### Assessment Reactivity

One of the major methodological issues facing SBI research in general is the degree to which simply being part of a study that assesses alcohol use may affect study results (Elbourne 2014; Finney 2008; McCambridge and Kypri 2011; McCambridge et al. 2014). Indeed, studies find that simply evaluating people’s drinking—as would happen in the screening part of an SBI—has a robust effect on drinking behavior over time (Dearing et al. 2013; Epstein et al. 2005). This “assessment reactivity” may underlie the similar changes in both the intervention and control groups seen among many of the studies reviewed here. To reduce the potential for assessment reactivity, future randomized controlled studies could include an additional minimal-assessment control arm that only measures outcomes at the final followup.

## Summary

There is robust evidence that in-person alcohol SBIs are effective when delivered to patients by staff in medical settings (Moyer 2013; Newton et al. 2013; O’Donnell et al. 2014). However, the implementation rates of these face-to-face SBIs remain suboptimal (Hingson et al. 2013; McKnight-Eily et al. 2014). Technology-based solutions, such as computerized SBI systems, may help to address this problem, but evidence for their effectiveness is less clear. This review found a burgeoning, but still small, research field with only 23 published papers representing 18 different trials evaluating the use of technology-based alcohol SBIs among adults, pregnant women, and adolescents in medical settings. The studies all found that technology-based alcohol SBIs are feasible for delivery in the medical setting and acceptable among patients, but most had methodological limitations. Only 13 of the 18 were controlled trials, and the majority were conducted in adult populations, with just four conducted among adolescents and only two among pregnant women. More than half of the studies took place in EDs, which offers a prime “teachable” moment, particularly for injured patients. However, more studies are needed in primary care and other ambulatory medical care settings, where patients may have periodic and ongoing contact with their health care providers. Such longitudinal patient–clinician relationships would allow for continued support and followup regarding recommended behavior changes. New studies also will benefit from bigger sample sizes to increase the power of their findings, more comprehensive participant recruitment, higher retention rates, and longer follow-up periods.

Finally, a promising new direction for the field would be to evaluate the potential of mobile technologies that can be used in medical settings. Suffoletto and colleagues (2012) demonstrated that mobile devices offer the potential to act as “clinician-extenders,” allowing clinicians to support and interact with patients after a visit and potentially boost the effect of a computerized brief intervention delivered in the medical setting. A review by Heron and Smyth (2010) of studies examining the use of ecological momentary interventions delivered through mobile technology, such as cell phones and tablet computers, found them to be feasible and acceptable and show efficacy for addressing a variety of psychosocial and other health behaviors, including alcohol use. Research also may begin to emerge on the use of smart-phone apps and social-networking sites like Facebook for underage drinking prevention and intervention.

## Figures and Tables

**Table 1:** Characteristics of Computer-Assisted Alcohol Screening and Brief Intervention (SBI) Studies Conducted in Health Care Settings

Authors (Year)	Study Population	Setting	Screening and Other Measures	Study Design/Treatment Conditions	Follow-up Period (%Completed)	Results
**Adults (Age 18 or Older): Primary Care**						
Butler et al. (2003)	English- or Spanish-speaking primary care patients (ages 18–99, *N* = 2,053 screened, 128 screened positive and completed followup, 68% female)	Primary care practices in Massachusetts, New York, and Florida	Alcohol Use Disorders Identification Test (AUDIT) Stage-of-change measure	Before-and-after, each site own control: Control phase (*N* = 66): Standard care with AUDIT after visit Treatment phase (*N* = 62): 20-minute computerized SBI completed in medical office before visit, with tailored feedback and information to reduce risky drinking; clinician can be given printed report with suggested brief interventions	6 months (85%)	Spanish version had lower AUDIT+ detection rates than English version; no such difference found with traditional AUDIT. AUDIT-C scores declined for both groups during followup; no intervention effect; no difference between language groups.
Kypri et al. (2008)	University health service patients screening positive for at-risk drinking (ages 17–29; *N* = 975 screened, 429 screened positive, 52% female)	University health service in New Zealand	AUDIT Past-2-weeks alcohol consumption Alcohol Problems Scale	Randomized controlled trial (RCT) three groups: Single-dose 10-minute Web-based SBI (*N* = 138): Assessment, personalized normative feedback, risk status, comparison of consumption with recommended limits Multi-dose Web-based SBI (*N* = 145): same as above repeated at 1 and 6 months Control (*N* = 146): Information pamphlet only	6 months (84%) 12 months (84%)	Both intervention groups had lower alcohol consumption, AUDIT scores, and alcohol problems at 6 and 12 months compared with the control group. Single-dose and multi-dose effects similar; provision of up to two additional sessions did not increase efficacy.
Bendtsen et al. (2011)	Primary care patients with risky drinking (ages 18 or older; *N* = 7,863 screened, 3,169 screened positive, 578 received e-SBI, 347 completed followup, 41% female)	Primary care clinics in one Swedish county	Average weekly use Heavy episodic drinking (HED) occasions per month	Observational study of two cohorts: “Self-referred” (*N* = 139): computerized SBI in clinic completed on own initiative “Staff-referred”: (*N* = 208) invited by clinician to complete computerized SBI after visit Behavioral intervention (BI) for both was printout of personalized written feedback	3 months (60%)	No significant between-group differences at baseline and 3 months. “Staff-referred” had reduction in weekly alcohol use but “self-referred” did not. Significant reduction in HED for both. Follow-up responders more likely to be older, have lower weekly alcohol use at baseline than non-responders; no difference in HED.
Cucciare et al. (2013)	Military veterans screening positive for alcohol misuse (*N* = 167, 12% female)	Veterans Affairs primary care clinics in California	AUDIT-C Timeline Follow-Back Alcohol-related consequences	RCT two groups: Intervention (*N* = 89): Standard care plus Web-based 10-minute SBI with assessment, personalized normative feedback, education, summary of alcohol-related consequences and risk factors, and self-reported motivation to change Control (*N* = 78): Standard care only (brief counseling by PCP)	3 months (86%) 6 months (84%)	Alcohol consumption and severity of alcohol-related problems declined for both groups. No differences between groups.
**Adults (Age 18 or Older): Emergency Department (ED)**						
Karlsson and Bendtsen (2005)	ED patients (ages 18–70, *N* = 44, % female not available)	ED of university hospital in Sweden	Modified AUDIT-C Patients’ ratings of computerized screening and personalized feedback	Single-group acceptability study: Computerized screening and printout of personalized feedback and advice given to patient	N/A	95% rated computer easy to use. 67% rated being screened positively. 76% rated feedback and advice printout positively. 74% preferred printout over nurse or doctor delivery. 93% would read advice.
Blow et al. (2006)	Sub-critically injured ED patients screening positive for at-risk drinking (ages 19 or older, *N* = 4,476 screened, 577 screened positive and received BI, 29% female)	Midwestern level 1 trauma center in university hospital	Frequency of alcohol consumption and HED in past 3 months Drinker Inventory of Consequence— Short Inventory of Problems	RCT four groups: Computerized screening plus computer generated: Tailored message booklet with clinician-delivered brief advice (*N* = 129) Tailored message booklet only (*N* = 121) Generic message booklet with advice (*N* = 124) Generic message booklet only (*N* = 120)	3 months (86%) 12 months (86%)	All groups reduced mean drinks per week, HED, and alcoholrelated consequences by 12 months. No difference in outcomes between tailored vs. generic message conditions. Brief advice had greater reductions than no advice, particularly among females and those aged 22 and older.
Neumann et al. (2006)	Sub-critically injured ED patients screening positive for at-risk drinking (ages 18 or older, *N* = 1,139, 79% female)	ED in Germany	AUDIT Readinessto-Change questionnaire Percent of patients with at-risk drinking (more than 30 g/d men; more than 20 g/d women)	RCT two groups: Intervention: Standard care plus computerized SBI (*N* = 561): with customized normative feedback, advice, change strategies, and summary letter printed for patient before ED discharge Control (*N* = 575): Standard care only	6 months (63%) 12 months (58%)	Significant intervention effects at 6 and 12 months: intervention group had lower percent of patients reporting at-risk drinking, and greater decrease in alcohol intake, compared with control subjects.
Nilsen et al. (2009) Trinks et al. (2010)	ED patients screening positive for risky drinking (ages 18–69, *N* = 1,570 screened, 560 screened positive and received BI, 93 completed followup, 39% female)	County hospital ED in Sweden	AUDIT-C	RCT two groups: Computerized screening with printout given to patient of: “Long-feedback” (*N* = 52): Traffic light graphic with risk level (hazardous, elevated, or no risk) and other tailored feedback about drinking pattern, and information to enhance motivation to change behavior “Short-feedback” (*N* = 41): Traffic light graphic only	6 months (17%)	41% of those requested to do computer SBI did. Both groups had reduced weekly alcohol consumption and HED frequency at 6 months. No differences in change over time between groups. 6-month respondents had lower HED frequency at baseline than non-respondents.
Vaca et al. (2011)	English- or Spanish-speaking ED patients (ages 18–65 or older, *N* = 4,375 screened, 742 screened positive and received BI, 385 consented to follow-up, 35% female)	University hospital ED in California	AUDIT Drinks per week	Single-cohort observational study: Intervention: Computerized SBI involving brief negotiated interview, and personal alcohol reduction plans	6 months (57%)	47% of at-risk drinkers reduced drinking to below NIAAA-recommended limits. Decreased frequency of driving while impaired. Reductions greater among those with AUDIT scores higher than 8.
Suffoletto et al. (2012)	ED patients (ages 18–24; *N* = 109, 52 screened positive, 45 consented to participate, 64% female)	Urban EDs in Pennsylvania	AUDIT Timeline Follow-Back	RCT three groups: Intervention (*N* = 15): Weekly text message (TM) feedback with goal setting Assessment only (*N* = 15): Weekly TM-based assessments, no feedback Control (*N* = 15): Weekly TM notifying number of weeks until 3-month followup	3 months (86%)	93% of intervention and assessment groups replied *one or more times* to weekly TM queries about drinking; 80% of intervention group replied to all 12 weeks of queries. Intervention reduced heavy-drinking days and drinks per drinking day more than assessment-only.
Murphy et al. (2013)	ED patients (ages 21–85 years, *N* = 517, 63% female)	ED of urban academic medical center in New York	AUDIT Patient acceptance and comprehension questionnaire Research staff questionnaire	Single-group feasibility study: 15-minute Web-based SBI with assessment, tailored risk-level education, customized normative feedback, list of local alcohol treatment agencies	N/A	98% completed CASI program. 89% liked program. 93% found it easy to use. 90% accurately reported alcohol risk level after program completion.
**Adults (Age 18 or Older): Hospital Outpatient Clinics**						
Johnson et al. (2013)	Hospital outpatients (ages 18 or older, *N* = 99 completed SBI, 69 invited for followup, 46% female)	Hospital ambulatory care center in Australia	AUDIT Peak blood alcohol concentration (BAC) Leeds Dependence Questionnaire History of Trauma scale	Single-group feasibility study: Computerized SBI with normative feedback on screening results and peak BAC, comparison to recommended limits (not shown for low-risk drinkers), information about health and behavioral risks of different BACs, estimate of spending on alcohol per month, tips for reducing risk and local treatment options	Within few days of visit (75%)	93% of eligible consenting patients completed SBI. 94% found it easy to complete. 95% reported responding honestly. 80% found feedback useful. 96% had no concern about privacy.
**Pregnant Women**						
Tzilos et al. (2011)	Pregnant women screening positive for problem alcohol use (ages 18–45, *N* = 50)	Urban prenatal care clinic in Michigan	T-ACE Timeline FollowBack Readiness to Change Acceptability of software Birth outcome variables	RCT two groups: Intervention (*N* = 27): 15-to 20-minute computerized SBI with educational content tailored to pregnant women, and to their current drinking status and motivation to change Control (*N* = 23): Questionnaire on television show preferences and shown videos of popular shows	1 month (96%)	High acceptability of computerized screening and BI. Both groups showed significant decline in reported alcohol consumption during followup; no differ-ences between groups. Babies born to BI group had significantly higher birth weight compared with control subjects.
Pollick et al. (2013)	Pregnant African-American women who screened positive for problem drinking but quit during pregnancy (ages 18–29, *N* = 18)	Urban prenatal care clinic in Michigan	T-ACE Alcohol use Acceptability of software Semistructured interview about user experience	Single-group pretesting study Computerized SBI: 20minute interactive tailored program with content based on MI techniques with normed feedback, decisional balance exercise, menu of change (or relapse prevention) options, referral to local treatment options	N/A	High ratings for software approval, ease of use, and perceived helpfulness. Videos and graphs/charts rated most useful components.
**Adolescents (Age 17 or Younger)**						
Gregor et al. (2003) Maio et al. (2005)	ED patients with minor injuries (ages 14–18 years, *N* = 655, 33% female)	ED of academic medical centers in Michigan	Alcohol Misuse Index of negative consequences of alcohol use Binge-drinking episodes in past 3 months Driving after drinking or riding with a driver that had been drinking	RCT two groups: Intervention (*N* = 329): Computerized screening and single-session BI interactive educational program (virtual house party) to increase knowledge about risks, enhance refusal skills, decrease intention to use Control (*N* = 326): Baseline survey with standard care only	3 months (93%) 12 months (89%)	Overall sample 94% liked program, 74% reported it made them rethink their alcohol use, 5% needed assistance to use it. No differences in alcohol outcomes between intervention and control: both decreased from baseline to 3 months, but returned to baseline levels by 12 months. Subgroup with baseline drinking and driving Alcohol misuse and binge drinking lower at 12 months in intervention group.
Cunningham et al. (2009, 2012) Walton et al. (2010)	ED patients with past-year violence and alcohol use (ages 14–18, *N* = 3,338 screened, 726 screened positive and consented to study, 56% females)	Urban ED in Michigan	AUDIT-C POSIT Conflict Tactic scale Violence consequences	RCT three groups: Computerized BI (*N* = 237) Therapist-delivered BI (*N* = 254) Both 35 minutes and based on motivational interviewing, with normative feedback and skills training Control (*N* = 235): standard care with community resource brochure (also given to BI groups)	3 months (86%) 6 months (86%) 12 months (84%)	3 months: computer and therapist BI groups showed similar significant reductions in positive alcohol and violence attitudes, increases in refusal self-efficacy. 6 months: Both BI groups less likely to report alcohol-related consequences than control group, but no effect on drinking frequency. 12 months: significant therapist-BI effect on peer aggression and victimization; no BI effect (computer or therapist) on any alcohol variables.
Harris et al. (2012) Louis-Jacques et al. (2014)	Primary care patients (ages 12–18, *N* = 2,092 in United States [USA], 589 in Czech Republic [CZR]; USA/CZR 57%/47% females)	Primary care clinics in New England, and Prague, Czech Republic	CRAFFT screener Timeline Follow-Back Postvisit questionnaire Personal Consequences Scale	Before-and-after, each site own control: Control phase (USA/CZR *N* = 1,068/297): Treatment as usual (TAU) Intervention phase (USA/CZR *N* = 1,028/292): 10-minute computer-assisted screening and provider brief advice (cSBA) with screening, risk-level feedback, educational pages, and provider report with screen results and prompts for 2 to 3 minutes of counseling	3 months (73%/88%) 12 months (73%/90%)	3 months: cSBA significantly reduced alcohol use rates compared with TAU in USA sample but not in CZR sample. Larger cSBA cessation effect found among drinking youth with peer risk (having friends who drank). 12 months: cSBA effect attenuated but still significant among New England youth.
Walton et al. (2014)	ED patients screening positive for risky drinking (ages 14–20, *N* = 4,389 screened, 1,053 screened positive, 836 consented to study, 48% female)	Urban ED in Michigan	AUDIT-C Alcohol-related consequences (RAPI) Psychological constructs related to behavior change: – Importance of cutting back – Likelihood to cut down in next 30 days – Readiness to stop – Desire for help to cut down	RCT three groups: Computerized BI (*N* = 252): Offline “Facebook”-styled program Therapist-delivered BI (*N* = 256) – Both BI had tailored normative feedback, based on motivational interviewing and cognitive–behavioral strategies Control (*N* = 281): Standard care with community resource brochure (also given to BI groups)	Immediate posttest (99%)	Increased importance of change in both BI groups compared with control groups. Increased readiness to stop in Therapist BI group. BI components positively related to changes in psychological constructs: Computer BI – Benefits of change – Alternate activities – Choosing goal to reduce or stop Both – Tools for reducing or stopping use – Personal strengths review

NOTES: Abbreviations: AUDIT-C: Alcohol Use Disorders Identification Test—Consumption items (items 1–3) CASI: Computerized alcohol screening and intervention CRAFFT: Car, Relax, Alone, Forget, Family/Friends, Trouble PCP: Primary care provider POSIT: Problem Oriented Screening Instrument for Teenagers RAPI: Rutgers Alcohol Problem Index T-ACE: Mnemonic for 4-item screener for problem alcohol use (Tolerance, Annoyed, Cut down, Eye-opener)

## References

[b1-arcr-36-1-63] American Academy of Pediatrics Committee on Substance Abuse. (2011). Substance use screening, brief intervention, and referral to treatment for pediatricians. Pediatrics.

[b2-arcr-36-1-63] Anand V, Carroll AE, Downs SM (2012). Automated primary care screening in pediatric waiting rooms. Pediatrics.

[b3-arcr-36-1-63] Babor T, Higgins-Biddle J (2001). Brief Interventions for Hazardous and Harmful Drinking: A Manual for Use in Primary Care.

[b4-arcr-36-1-63] Bandura A (1977). Self-efficacy: Toward a unifying theory of behavioral change. Psychological Review.

[b5-arcr-36-1-63] Barnett AG, van der Pols JC, Dobson AJ (2005). Regression to the mean: What it is and how to deal with it. International Journal of Epidemiology.

[b6-arcr-36-1-63] Beck F, Guignard R, Legleye S (2014). Does computer survey technology improve reports on alcohol and illicit drug use in the general population? A comparison between two surveys with different data collection modes in France. PloS One.

[b7-arcr-36-1-63] Bendtsen P, Stark Ekman D, Johansson A (2011). Referral to an electronic screening and brief alcohol intervention in primary health care in Sweden: Impact of staff referral to the computer. International Journal of Telemedicine and Applications.

[b8-arcr-36-1-63] Blow FC, Barry KL, Walton MA (2006). The efficacy of two brief intervention strategies among injured, at-risk drinkers in the emergency department: Impact of tailored messaging and brief advice. Journal of Studies on Alcohol.

[b9-arcr-36-1-63] Blow FC, Ilgen MA, Walton MA (2009). Severity of baseline alcohol use as a moderator of brief interventions in the emergency department. Alcohol and Alcoholism.

[b10-arcr-36-1-63] Bush K, Kivlahan DR, McDonell MB (1998). The AUDIT alcohol consumption questions (AUDIT-C): An effective brief screening test for problem drinking. Ambulatory Care Quality Improvement Project (ACQUIP). Alcohol Use Disorders Identification Test. Archives of Internal Medicine.

[b11-arcr-36-1-63] Butler SF, Chiauzzi E, Bromberg JI (2003). Computer-assisted screening and intervention for alcohol problems in primary care. Journal of Technology in Human Services.

[b12-arcr-36-1-63] Butler SF, Villapiano A, Malinow A (2009). The effect of computer-mediated administration on self-disclosure of problems on the addiction severity index. Journal of Addiction Medicine.

[b13-arcr-36-1-63] Carey KB, Scott-Sheldon LAJ, Elliott JC (2012). Face-to-face versus computer-delivered alcohol interventions for college drinkers: A meta-analytic review, 1998 to 2010. Clinical Psychology Review.

[b14-arcr-36-1-63] Chang G (2002). Brief interventions for problem drinking and women. Journal of Substance Abuse Treatment.

[b15-arcr-36-1-63] Chisolm DJ, Gardner W, Julian T, Kelleher KJ (2008). Adolescent satisfaction with computer-assisted behavioral risk screening in primary care. Child and Adolescent Mental Health.

[b16-arcr-36-1-63] Cucciare MA, Weingardt KR, Ghaus S (2013). A randomized controlled trial of a web-delivered brief alcohol intervention in Veterans Affairs primary care. Journal of Studies on Alcohol and Drugs.

[b17-arcr-36-1-63] Cunningham RM, Chermack ST, Zimmerman MA (2012). Brief motivational interviewing intervention for peer violence and alcohol use in teens: One-year follow-up. Pediatrics.

[b18-arcr-36-1-63] Cunningham RM, Walton MA, Goldstein A (2009). Three-month follow-up of brief computerized and therapist interventions for alcohol and violence among teens. Academic Emergency Medicine.

[b19-arcr-36-1-63] Dearing RL, Witkiewitz K, Connors GJ, Walitzer KS (2013). Prospective changes in alcohol use among hazardous drinkers in the absence of treatment. Psychology of Addictive Behaviors.

[b20-arcr-36-1-63] Donoghue K, Patton R, Phillips T (2014). The effectiveness of electronic screening and brief intervention for reducing levels of alcohol consumption: A systematic review and meta-analysis. Journal of Medical Internet Research.

[b21-arcr-36-1-63] Elliot DL, Hickam DH (1990). Use of the T-ACE questions to detect risk-drinking. American Journal of Obstetrics and Gynecology.

[b22-arcr-36-1-63] Epstein EE, Drapkin ML, Yusko D (2005). Is alcohol assessment therapeutic? Pretreatment change in drinking among alcohol-dependent women. Journal of Studies on Alcohol.

[b23-arcr-36-1-63] Finney JW (2008). Regression to the mean in substance use disorder treatment research. Addiction.

[b24-arcr-36-1-63] Gadomski AM, Fothergill KE, Larson S (2015). Integrating mental health into adolescent annual visits: Impact of previsit comprehensive screening on within-visit processes. Journal of Adolescent Health.

[b25-arcr-36-1-63] Gfroerer J, Wright D, Kopstein A (1997). Prevalence of youth substance use: The impact of methodological differences between two national surveys. Drug and Alcohol Dependence.

[b26-arcr-36-1-63] Harris S, Csémy L, Sherritt L (2012). Computer-facilitated substance use screening and brief advice for teens in primary care: An international trial. Pediatrics.

[b27-arcr-36-1-63] Harris SK, Csemy L, Sherritt L (2011). Screening and brief physician’s advice to reduce teens’ risk of substance-related car crashes: An international trial. Association for Medical Education and Research in Substance Abuse (AMERSA) Annual Conference.

[b28-arcr-36-1-63] Heron KE, Smyth JM (2010). Ecological momentary interventions: Incorporating mobile technology into psychosocial and health behaviour treatments. British Journal of Health Psychology.

[b29-arcr-36-1-63] Hester R, Miller W (2003). Handbook of Alcoholism Treatment Approaches: Effective Alternatives.

[b30-arcr-36-1-63] Hingson RW, Zha W, Iannotti RJ, Simons-Morton B (2013). Physician advice to adolescents about drinking and other health behaviors. Pediatrics.

[b31-arcr-36-1-63] Johnson NA, Kypri K, Attia J (2013). Development of an electronic alcohol screening and brief intervention program for hospital outpatients with unhealthy alcohol use. JMIR Research Protocols.

[b32-arcr-36-1-63] Jonas DE, Garbutt JC, Amick HR (2012). Behavioral counseling after screening for alcohol misuse in primary care: A systematic review and meta-analysis for the US Preventive Services Task Force. Annals of Internal Medicine.

[b33-arcr-36-1-63] Kadden R, Carroll K, Donovan D (1995). Cognitive-behavioral coping skills therapy manual: A clinical research guide for therapists treating individuals with alcohol abuse and dependence. Project MATCH Monograph Series.

[b34-arcr-36-1-63] Kaner EF, Beyer F, Dickinson HO (2007). Effectiveness of brief alcohol interventions in primary care populations. Cochrane Database of Systematic Reviews.

[b35-arcr-36-1-63] Kaner EFS, Dickinson HO, Beyer F (2009). The effectiveness of brief alcohol interventions in primary care settings: A systematic review. Drug and Alcohol Review.

[b36-arcr-36-1-63] Karlsson A, Bendtsen P (2005). Acceptability of a computerized alcohol screening and advice routine in an emergency department setting: A patient perspective. Addictive Behaviors.

[b37-arcr-36-1-63] Knight JR, Sherritt L, Shrier LA (2002). Validity of the CRAFFT substance abuse screening test among adolescent clinic patients. Archives of Pediatrics & Adolescent Medicine.

[b38-arcr-36-1-63] Kypri K, Langley JD, Saunders JB (2008). Randomized controlled trial of web-based alcohol screening and brief intervention in primary care. Archives of Internal Medicine.

[b39-arcr-36-1-63] Louis-Jacques J, Knight JR, Sherritt L (2014). Do risky friends change the efficacy of a primary care brief intervention for adolescent alcohol use?. Journal of Adolescent Health.

[b40-arcr-36-1-63] Madden M, Lenhart A, Duggan M (2013). Teens and Technology.

[b41-arcr-36-1-63] Maio RF, Shope JT, Blow FC (2005). A randomized controlled trial of an emergency department-based interactive computer program to prevent alcohol misuse among injured adolescents. Annals of Emergency Medicine.

[b42-arcr-36-1-63] Marsch LA, Bickel WK, Grabinski MJ (2007). Application of interactive, computer technology to adolescent substance abuse prevention and treatment. Adolescent Medicine: State of the Art Reviews.

[b43-arcr-36-1-63] McCambridge J, Kypri K (2011). Can simply answering research questions change behaviour? Systematic review and meta analyses of brief alcohol intervention trials. PloS One.

[b44-arcr-36-1-63] McCambridge J, Witton J, Elbourne DR (2014). Systematic review of the Hawthorne effect: New concepts are needed to study research participation effects. Journal of Clinical Epidemiology.

[b45-arcr-36-1-63] McKnight-Eily LR, Liu Y, Brewer RD (2014). Vital signs: Communication between health professionals and their patients about alcohol use: 44 states and the District of Columbia, 2011. MMWR: Morbidity and Mortality Weekly Report.

[b46-arcr-36-1-63] McNeely J, Strauss SM, Wright S (2014). Test-retest reliability of a self-administered alcohol, smoking and substance involvement screening test (ASSIST) in primary care patients. Journal of Substance Abuse Treatment.

[b47-arcr-36-1-63] Miller WR, Rollnick S (2012). Motivational Interviewing: Helping People Change, Third Edition.

[b48-arcr-36-1-63] Mitchell SG, Gryczynski J, Grady KE, Schwartz RP (2013). SBIRT for adolescent drug and alcohol use: Current status and future directions. Journal of Substance Abuse Treatment.

[b49-arcr-36-1-63] Mitchell S, Gryczynski J, Gonzales A (2012). Screening, brief intervention, and referral to treatment (SBIRT) for substance use in a school-based program: Services and outcomes. American Journal on Addictions.

[b50-arcr-36-1-63] Moyer VA, Preventive Services Task Force. (2013). Screening and behavioral counseling interventions in primary care to reduce alcohol misuse: U.S. Preventive Services Task Force recommendation statement. Annals of Internal Medicine.

[b51-arcr-36-1-63] Murphy MK, Bijur PE, Rosenbloom D (2013). Feasibility of a computer-assisted alcohol SBIRT program in an urban emergency department: Patient and research staff perspectives. Addiction Science & Clinical Practice.

[b52-arcr-36-1-63] Murray E, White IR, Varagunam M (2013). Attrition revisited: Adherence and retention in a web-based alcohol trial. Journal of Medical Internet Research.

[b53-arcr-36-1-63] Naimi TS, Cole TB (2014). Electronic alcohol screening and brief interventions: Expectations and reality. JAMA.

[b54-arcr-36-1-63] National Center on Addiction and Substance Abuse (CASA) (2011). Adolescent Substance Use: America’s # 1 Public Health Problem.

[b55-arcr-36-1-63] National Institute on Alcohol Abuse and Alcoholism (NIAAA) (1995). Helping Patients Who Drink Too Much: A Clinician’s Guide.

[b56-arcr-36-1-63] National Institute on Alcohol Abuse and Alcoholism (NIAAA) (2010). Rethinking Drinking: Alcohol and Your Health.

[b57-arcr-36-1-63] National Institute on Alcohol Abuse and Alcoholism (NIAAA) (2013). Program Announcement: Implications of New Digital Media Use for Underage Drinking, Drinking-Related Behaviors, and Prevention Research (R01).

[b58-arcr-36-1-63] Neumann T, Neuner B, Gentilello LM (2004). Gender differences in the performance of a computerized version of the Alcohol Use Disorders Identification Test in subcritically injured patients who are admitted to the emergency department. Alcohol: Clinical and Experimental Research.

[b59-arcr-36-1-63] Neumann T, Neuner B, Weiss-Gerlach E (2006). The effect of computerized tailored brief advice on at-risk drinking in subcritically injured trauma patients. Journal of Trauma.

[b60-arcr-36-1-63] Newton AS, Dong K, Mabood N (2013). Brief emergency department interventions for youth who use alcohol and other drugs: A systematic review. Pediatric Emergency Care.

[b61-arcr-36-1-63] Nilsen P, Festin K, Guldbrandsson K (2009). Implementation of a computerized alcohol advice concept in routine emergency care. International Emergency Nursing.

[b62-arcr-36-1-63] O’Donnell A, Anderson P, Newbury-Birch D (2014). The impact of brief alcohol interventions in primary healthcare: A systematic review of reviews. Alcohol and Alcoholism.

[b63-arcr-36-1-63] Olson AL, Gaffney CA, Hedberg VA, Gladstone GR (2009). Use of inexpensive technology to enhance adolescent health screening and counseling. Archives of Pediatrics & Adolescent Medicine.

[b64-arcr-36-1-63] Ondersma SJ, Grekin ER, Svikis D (2011). The potential for technology in brief interventions for substance use, and during-session prediction of computer-delivered brief intervention response. Substance Use & Misuse.

[b65-arcr-36-1-63] Ozer EM, Adams SH, Lustig JL (2005). Increasing the screening and counseling of adolescents for risky health behaviors: A primary care intervention. Pediatrics.

[b66-arcr-36-1-63] Palfai TP, Zisserson R, Saitz R (2011). Using personalized feedback to reduce alcohol use among hazardous drinking college students: The moderating effect of alcohol-related negative consequences. Addictive Behaviors.

[b67-arcr-36-1-63] Perlis TE, Des Jarlais DC, Friedman SR (2004). Audio-computerized self-interviewing versus face-to-face interviewing for research data collection at drug abuse treatment programs. Addiction.

[b68-arcr-36-1-63] Pew Research Center’s Internet and American Life Project Internet User Demographics.

[b69-arcr-36-1-63] Pilowsky DJ, Wu LT (2013). Screening instruments for substance use and brief interventions targeting adolescents in primary care: A literature review. Addictive Behaviors.

[b70-arcr-36-1-63] Pollick SA, Beatty JR, Sokol RJ (2013). Acceptability of a computerized brief intervention for alcohol among abstinent but at-risk pregnant women. Substance Abuse.

[b71-arcr-36-1-63] Postel MG, de Haan HA, ter Huurne ED (2011). Attrition in web-based treatment for problem drinkers. Journal of Medical Internet Research.

[b72-arcr-36-1-63] Reinert DF, Allen JP (2002). The Alcohol Use Disorders Identification Test (AUDIT): A review of recent research. Alcoholism: Clinical and Experimental Research.

[b73-arcr-36-1-63] Rooke S, Thorsteinsson E, Karpin A (2010). Computer-delivered interventions for alcohol and tobacco use: A meta-analysis. Addiction.

[b74-arcr-36-1-63] Saitz R (2010). Alcohol screening and brief intervention in primary care: Absence of evidence for efficacy in people with dependence or very heavy drinking. Drug and Alcohol Review.

[b75-arcr-36-1-63] Sokol RJ, Martier SS, Ager JW (1989). The T-ACE questions: Practical prenatal detection of risk drinking. American Journal of Obstetrics and Gynecology.

[b76-arcr-36-1-63] Spirito A, Monti PM, Barnett NP (2004). A randomized clinical trial of a brief motivational intervention for alcohol-positive adolescents treated in an emergency department. Journal of Pediatrics.

[b77-arcr-36-1-63] Stevens J, Kelleher KJ, Gardner W (2008). Trial of computerized screening for adolescent behavioral concerns. Pediatrics.

[b78-arcr-36-1-63] Substance Abuse and Mental Health Services Administration (2011). White Paper on Screening, Brief Intervention and Referral to Treatment (SBIRT) in Behavioral Healthcare.

[b79-arcr-36-1-63] Suffoletto B, Callaway C, Kristan J (2012). Text-message-based drinking assessments and brief interventions for young adults discharged from the emergency department. Alcoholism: Clinical and Experimental Research.

[b80-arcr-36-1-63] Thomas BA, McCambridge J (2008). Comparative psychometric study of a range of hazardous drinking measures administered online in a youth population. Drug and Alcohol Dependence.

[b81-arcr-36-1-63] Trinks A, Festin K, Bendtsen P, Nilsen P (2010). Reach and effectiveness of a computer-based alcohol intervention in a Swedish emergency room. International Emergency Nursing.

[b82-arcr-36-1-63] Turner CF, Ku L, Rogers SM (1998). Adolescent sexual behavior, drug use, and violence: Increased reporting with computer survey technology. Science.

[b83-arcr-36-1-63] Tzilos GK, Sokol RJ, Ondersma SJ (2011). A randomized phase I trial of a brief computer-delivered intervention for alcohol use during pregnancy. Journal of Women’s Health.

[b84-arcr-36-1-63] Vaca FE, Winn D, Anderson CL (2011). Six-month follow-up of computerized alcohol screening, brief intervention, and referral to treatment in the emergency department. Substance Abuse.

[b85-arcr-36-1-63] Van Hook S, Harris SK, Brooks T (2007). The “Six T’s”: Barriers to screening teens for substance abuse in primary care. Journal of Adolescent Health.

[b86-arcr-36-1-63] Wachtel T, Staniford M (2010). The effectiveness of brief interventions in a clinical setting in reducing alcohol misuse and binge drinking in adolescents: A critical review of the literature. Journal of Clinical Nursing.

[b87-arcr-36-1-63] Walters ST, Neighbors C (2011). College prevention: A view of present (and future) web-based approaches. Alcohol Research & Health.

[b88-arcr-36-1-63] Walton MA, Chermack ST, Blow FC (2014). Components of brief alcohol interventions for youth in the emergency department. Substance Abuse.

[b89-arcr-36-1-63] Walton MA, Chermack ST, Shope JT (2010). Effects of a brief intervention for reducing violence and alcohol misuse among adolescents: A randomized controlled trial. JAMA.

[b90-arcr-36-1-63] White A, Kavanagh D, Stallman H (2010). Online alcohol interventions: A systematic review. Journal of Medical Internet Research.

[b91-arcr-36-1-63] Williams ML, Freeman RC, Bowen AM (2000). A comparison of the reliability of self-reported drug use and sexual behaviors using computer-assisted versus face-toface interviewing. AIDS Education and Prevention.

[b92-arcr-36-1-63] Wilson GB, Lock CA, Heather N (2011). Intervention against excessive alcohol consumption in primary health care: A survey of GPs’ attitudes and practices in England 10 years on. Alcohol and Alcoholism.

[b93-arcr-36-1-63] Wright DL, Aquilino WS, Supple AJ (1998). A comparison of computer-assisted paperand-pencil self-administered questionnaires in a survey on smoking, alcohol, and drug use. Public Opinion Quarterly.

[b94-arcr-36-1-63] Yuma-Guerrero PJ, Lawson KA, Velasquez MM (2012). Screening, brief intervention, and referral for alcohol use in adolescents: A systematic review. Pediatrics.

